# Implementation of an optimised tele-medicine platform for stroke in South Australia improves patient care

**DOI:** 10.3389/fneur.2024.1428198

**Published:** 2024-06-18

**Authors:** Craig Kurunawai, Chushuang Chen, Matthew Willcour, Aaron Tan, Joshua Mahadevan, Michael Waters, Jackson Harvey, Joanne Van Eunen, Karen Dixon, Bianca Piantedosi, Andrew Bivard, Mark William Parsons, Stephen M. Davis, Geoffrey Alan Donnan, Jim Jannes, Timothy Kleinig

**Affiliations:** ^1^Royal Adelaide Hospital, Adelaide, SA, Australia; ^2^Barossa Hills Fleurieu Local Health Network, Adelaide, SA, Australia; ^3^Faculty of Health and Medical Sciences, University of Adelaide, Adelaide, SA, Australia; ^4^South Western Sydney Clinical School, Faculty of Medicine, University of New South Wales, Liverpool, NSW, Australia; ^5^Ingham Institute of Applied Medical Research, Sydney, NSW, Australia; ^6^Flinders Medical Centre, Bedford Park, SA, Australia; ^7^Melbourne Brain Centre, University of Melbourne, Parkville, VIC, Australia; ^8^Department of Neurology, Royal Melbourne Hospital, Melbourne, VIC, Australia; ^9^Department of Neurology, Liverpool Hospital, Liverpool, SA, Australia

**Keywords:** Telestroke, tele-medecine, ischaemia stroke, thrombolys, thrombectomy

## Abstract

**Background:**

Patients with a large vessel occlusion require a transfer from a primary stroke centre to access thrombectomy, often over significant distances in regional areas. We sought to optimise stroke care access in the regional South Australian Tele-Strokeservice (SATS) to improve patient access to thrombectomy.

**Methods:**

We undertook a 24-month interventional historically controlled cohort study comparing acute stroke care metrics in the SATS. This consisted of a 12-month control period and a 12-month intervention monitoring period. The study intervention considered of an education package provided to the regional hospitals, a stroke neurologist roster to receive consultations and the intervention of a centralised tele-stroke system to provide treatment advice and organise patient transfers where needed. The SATS services 61 rural hospitals in South Australia, and Alice Springs in the Northern Territory. Suspected acute stroke patients presenting to the participating regional hospitals in SATS network where a telehealth consultation took place.

**Results:**

Over the study period, there were 919 patient referrals, with 449 consultations in the pre-intervention phase and 470 in the post-intervention phase. Demographic features in both epochs were similar. The post-intervention phase was associated with shorter door-to-scan time (35 min, IQR: 18,70; vs. 49 min, IQR:25,102, *p* < 0.0001), faster door-to-thrombolysis time (58 min, IQR: 39,91, vs.83 min, IQR: 55,100, *p* = 0.0324) and a higher portion of patients treated with thrombectomy (54, 11.5% vs. 26, 5.8%, *p* = 0.002).

**Conclusion:**

An optimised implementation of a streamlined telehealth platform with ongoing education and feedback to referring sites was associated with improved stroke workflow metrics and higher thrombectomy rates.

## Introduction

Stroke is the second leading cause of death and the most common cause of adult disability in the developed world (1). Stroke patients with a large vessel occlusion (LVO) face an especially devastating outcome due to the large volume of brain tissue affected, resulting in >50% of patients being dead or significantly disabled at 3 months without rapid and effective reperfusion treatment (2). The goal of acute ischemic stroke therapy is to either pharmacologically or mechanically remove the clot blocking the affected artery in order to restore blood flow to brain tissue that is ischaemic but still salvageable (the ischaemic penumbra). The efficacy of treatments for acute stroke has improved significantly over the last decade with the introduction of thrombectomy for patients with a large vessel occlusion and recent evidence supporting treatment for haemorrhage (3).

Stroke incidence is 20% higher in rural and remote areas (4), however the outcome of a patient presenting with a stroke to a regional hospital is associated with double the likelihood of significant lifelong disability than if a person were to have a stroke in a metropolitan area, likely from inequitable access to specialist services (5). Only 3% of patients in rural and remote areas of Australia have access to stroke specialists, compared to 77% of patients in metropolitan areas and treatment (6), if provided, is often considerably delayed for a condition where time to treatment is directly related to individual patient outcomes. In Australia, several tele-stroke services already exist and have reported success, such as the Victorian (7) and New South Wales tele-stroke services (8–10). However, much of Australia is un-covered with tele-stroke support despite evidence showing improved patient outcomes. There exist severe challenges in providing regional, low-volume stroke centres with adequate specialist cover and maintaining the local skillset where stroke presentations could make up as low as 1% of admissions. Importantly, telehealth services can often require significant costs to support the technology required in the remote consult.

Importantly, the clinical indication for thrombectomy has widened considerably since its introduction, allowing more patients access to this life-saving and quality of life improving therapy. This includes the expansion of eligibility up to 24 h post symptom onset and the trials indication that patients with a large established volume of infarct core still benefit from treatment. As a result, any patient with a large vessel occlusion ischaemic stroke anywhere in Australia is now a potential reperfusion candidate (11). However, thrombectomy treatment is only offered in large cities, with very limited access in regional centres. Therefore, patients presented in regional centres require transfer, often by air, to the thrombectomy centre. This makes the acute stroke care pathway for regional hospitals quite significant, and requires multi-agency coordination for patient retrieval. Optimised telecommunication and health consult software is a vital platform for accurate and efficient telemedicine services to support patient care both on-site and for transfer. In this study, we sought to compare the efficiency and treatment rate of stroke patients presenting to regional hospitals covered by the South Australian Tele-Stroke network over a 12-month period where a combined technical and education quality improvement intervention was undertaken, compared to 12 months of consecutive historical controls.

## Methods

### Materials and methods

**Figure 1:**
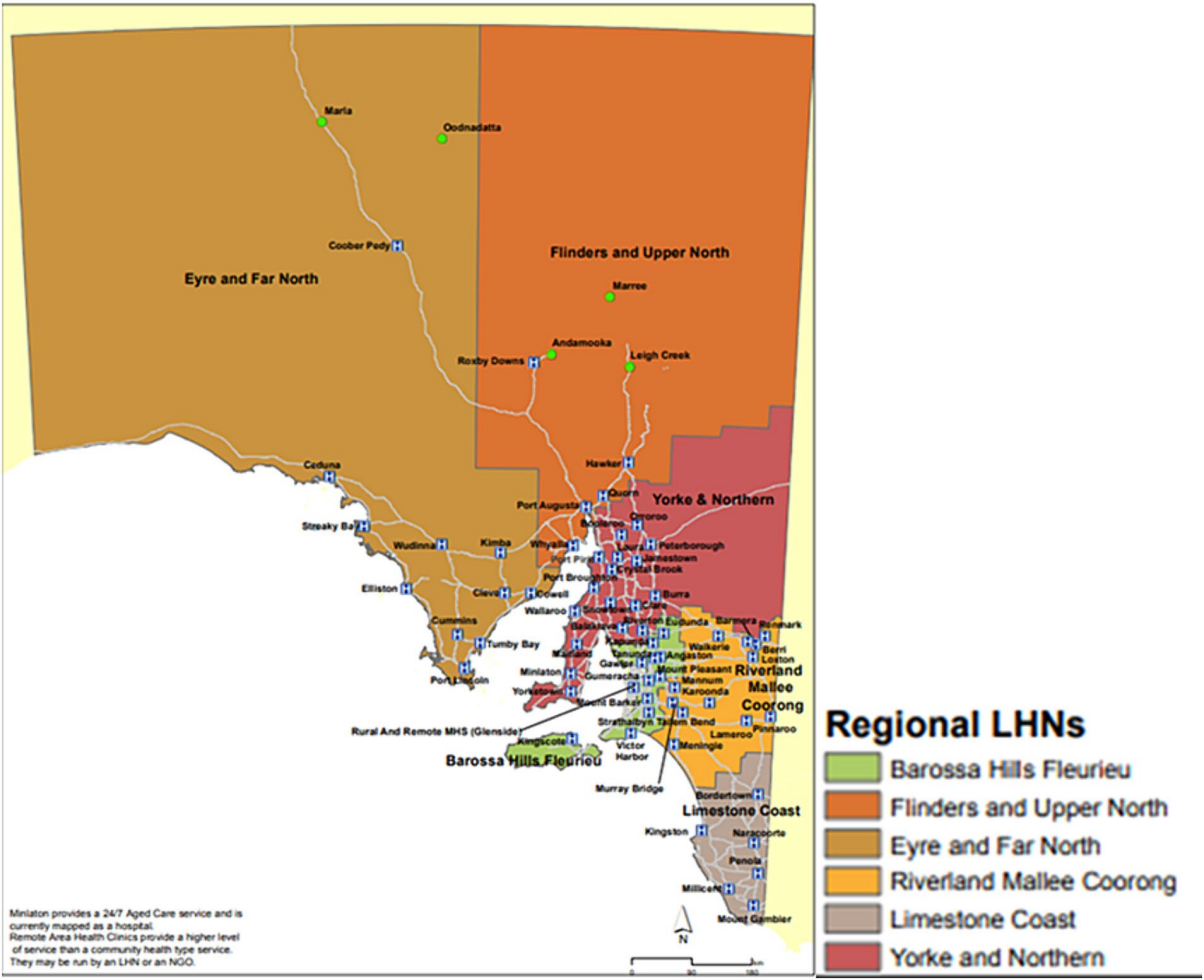
The South Australian Tele-Stroke Service (SATS) cover regional hospitals in South Australia and Alice Springs.

The South Australian Tele-Stroke Service (SATS) covers 61 hospitals in South Australia and the Northern Territory (Figure 1) and operates 24 h, 7 days a week, 365 days a year. Patients with symptoms of suspected stroke were assessed by ED clinical staff clinically with standard items such as the NIHSS and a medical history recorded, and where indicated or a standard computed tomography (CT) stroke imaging. Stroke specialist Neurologists on the SATS roster were contacted for patients potentially eligible for reperfusion following ‘code stroke pathways incorporating the Recognition of Stroke in the Emergency Room (ROSIER) Scale (12) on a dedicated toll-free telephone number. In tandem with neuroimaging initiation [non-contrast CT (NCCT), CT angiogram (CTA) +/− CT perfusion (CTP)] this then prompted a telemedicine consult by the Stroke specialist neurologist who would evaluate the patient via videoconference and communicate with the local hospital clinicians, receiving a patient handover and recommending treatment or transfer for thrombectomy where appropriate after reviewing the CT imaging.

We undertook a retrospective pre-post analysis of consecutive acute patients presenting to the regional hospitals in South Australia, where a telehealth consultation was provided by a central team of stroke-trained neurologists between January 2021 and December 2022. During this period, the number of sites covered and the standard stroke protocol was otherwise stable. This study sought to evaluate the impact of the dedicated telehealth platform “Zeus” and an education and ongoing quality improvement process as part of a broad intervention package. The study intervention was completed in December 2022.

During the study, a multidisciplinary stroke education program was continued for all clinical staff involved in both phases and is used for continuous feedback and quality improvement. The stroke education involved targeting rural and regional emergency department doctors and nurses in stroke care, as well as sharing expertise to improve stroke recognition, clinical assessments and scoring with the NIHSS, and process improvement options to treatment efficiency such as door-to-imaging or door-to-treatment times. During the post-intervention phase, the periodic data extracts enabled focused stroke metric feedback in both state-wide communities of practice forums that were held every 3 months. These forums would provide feedback on individual cases and identify pain points for discussion and solution mapping.

Additionally, monthly general sessions took place to provide ongoing feedback to sites around standard treatment metrics such as the number of patients assessed, the number of patients treated and time to imaging and treatment. The clinicians on the tele-stroke roster also met quarterly to review service data and share experiences to allow for improved communication to sites and between clinicians as a mechanism to enhance site feedback. Data collected pre and post-service implementation were standardised and aligned with clinical guidelines for acute stroke management.

We divided the analysis into two phases: pre-intervention from January to December 2021 and post-intervention of “Zeus” from January to December 2022. Clinical data were collected, including baseline National Institute of Health stroke scale (NIHSS), and pre-stroke modified Rankin Score (mRS). The date and time of stroke onset (or last date/time seen well), tele-stroke consult initiation, treatment decision, and time of thrombolysis were also collected. Door-to-scan was calculated as the time difference between patients’ arrival and the imaging, and door-to-thrombolytic time (DTT) was calculated as the difference between patient arrival and time of thrombolytic administration.

### Statistical analysis

Continuous data were assessed for normality using the Shapiro–Wilk method, with parametric data reported as mean (SD) and non-parametric data reported as median (IQR). Comparison of two groups was performed by the Student’s *t*-test (for parametric data) or the Wilcoxon Rank Sum test (for non-parametric data). For categorical variables, patient characteristics described as proportions and group comparison was conducted with Pearson Chi-squared test. Although comparisons between 2021 and 2022 data using the aforementioned tests for comparing two groups, differences between year-by-year data of the study period were tested using one-way analysis of variance (ANOVA) (parametric data) or Kruskal-Wallis (non-parametric) methodology. Data were analysed using STATA (V13.1, Stata Corp, College Station, Texas, United States), with alpha level set at 0.05.

## Results

For this study, there were 449 consultations in 40 regional hospitals performed in the pre-intervention phase (01/01/2021–31/12/2021) and 470 consultations in 52 hospitals performed in the post-intervention phase (01/01/2022–31/12/2022, Figures 2, 3). The median NIHSS of patients presenting in the pre-and post-intervention phase was similar (median 3, IQR: 1,7). The portion of patients with atrial fibrillation was similar between the pre-and post-intervention phases (77, 17.1% vs. 92, 19.6%, *p* = 0.394). The after-working-hours consultations were similar between the pre-and post-intervention phase (287, 63.9% vs. 293, 62.3%, *p* = 0.707, Table 1).

**Figure 2:**
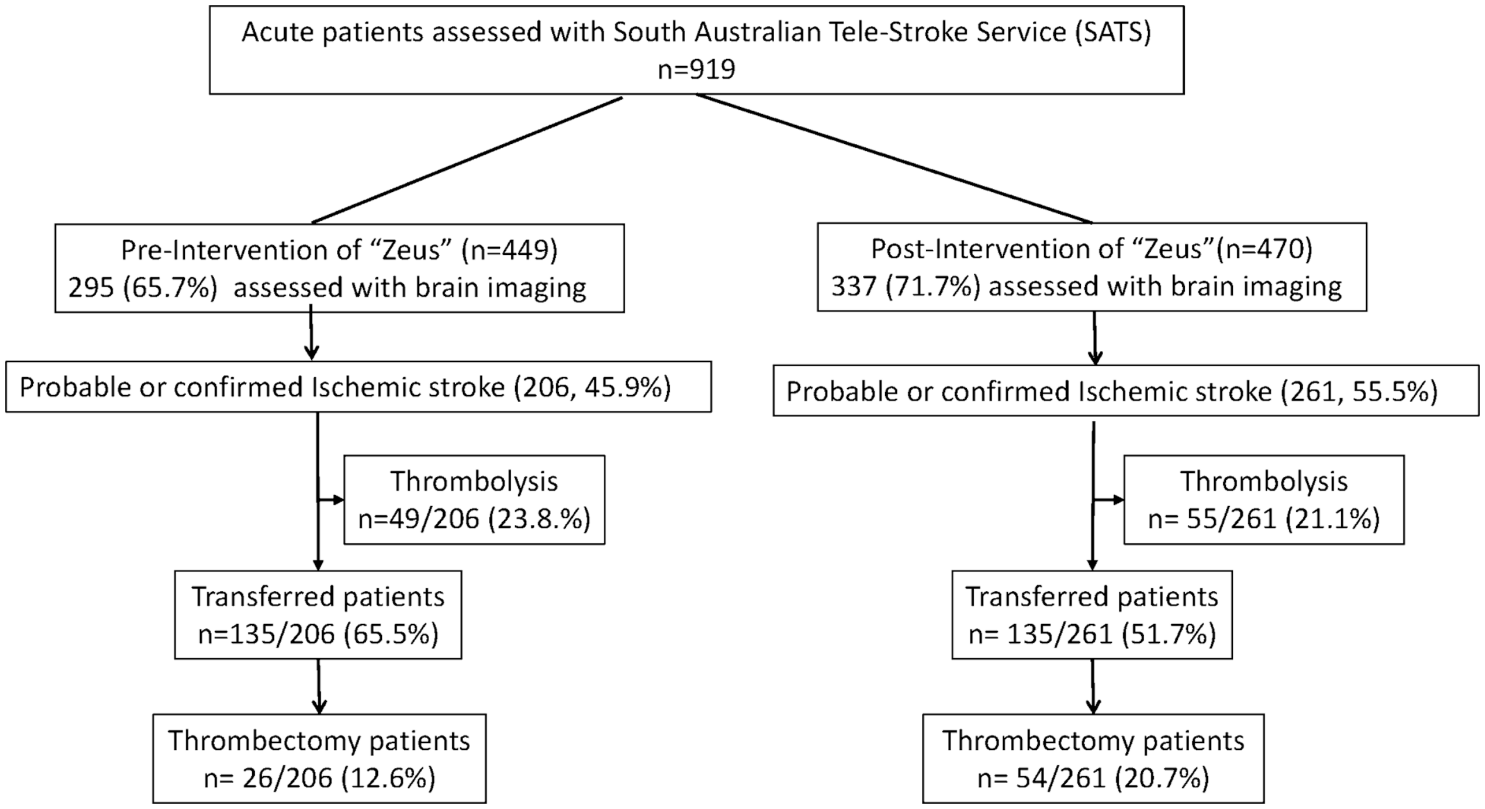
Diagram of patients in pre-and post-intervention phase.

**Figure 3:**
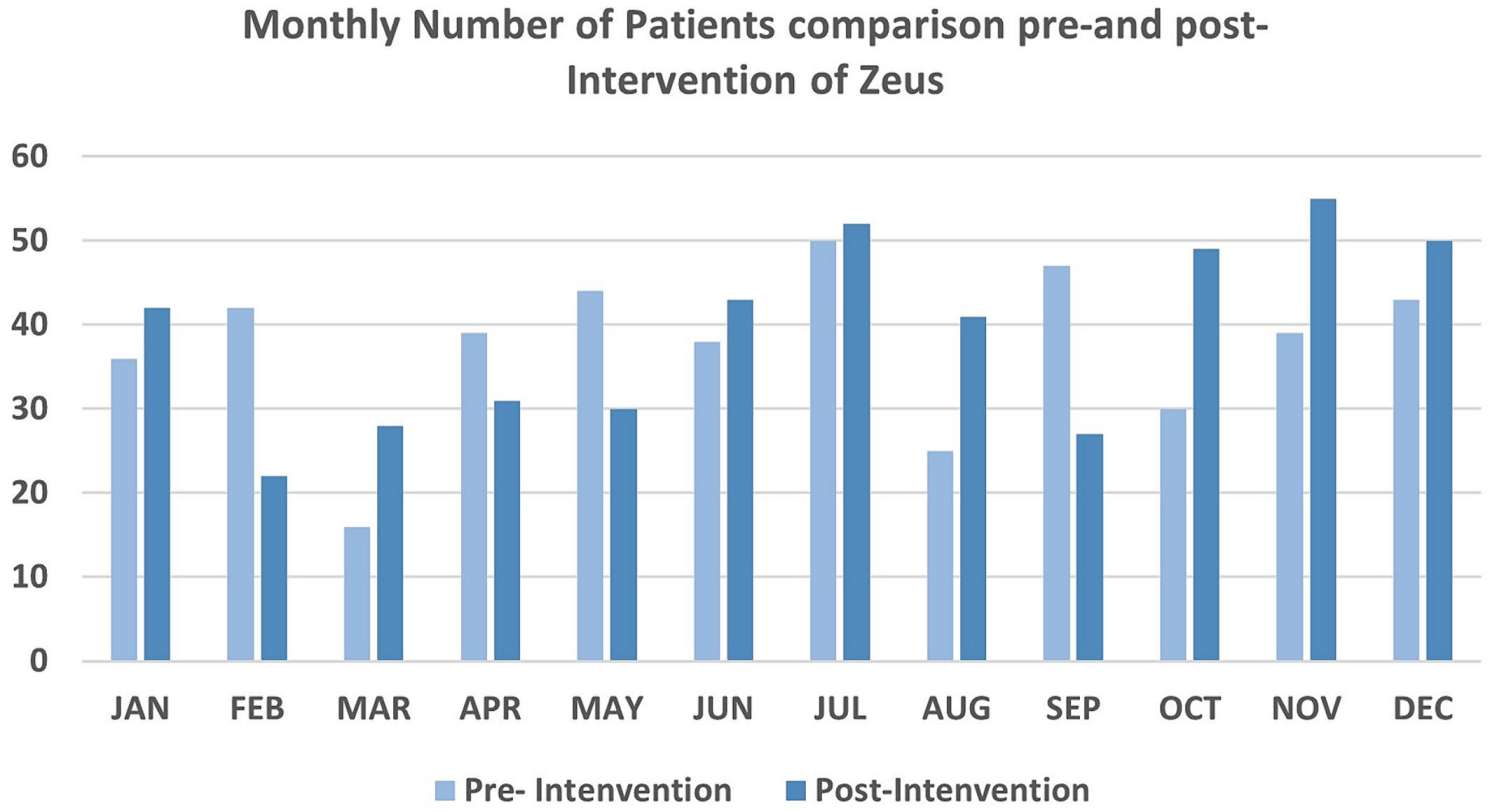
Number of patients using tele-stroke service comparison pre-and post-intervention in each month.

**Table 1:** Patient characteristics and workflow metrics of the South Australian Tele-Stroke Service (SATS).

	Pre-intervention (*n* = 449)	post-intervention (*n* = 470)	*p*
Baseline NIHSS (median, IRQ)	3 (1,7)	3 (1,7)	0.434
Pre-stroke mRS (0–2) (*n*, %)	408, 90.8%	428, 91.1%	0.907
AF (*n*, %)	77, 17.1%	92, 19.6%	0.394
After working hours consultations (*n*, %)	287, 63.9%	293, 62.3%	0.707
Consultation duration time (minutes) (median, range)	30 (10,110)	30 (10,120)	0.906
Patients had brain imaging (*n*, %)	295, 65.7%	337,71.7%	0.021
Probable or confirm of Ischemic stroke (*n*, %)	206, 45.9%	261, 55.5%	0.003
Thrombolysed patients (*n*, %)	49/206, 23.8%	55/261, 11.7%	0.484
Thrombectomy patients (*n*, %)	26/206, 12.6%	54/261, 20.7%	0.022
Door to scan (minutes) (median, IQR)	49 (25,102)	35 (18, 70)	<0.0001
Door to thrombolysis (minutes) (median, IQR)	83 (55,100)	58 (39,91)	0.032

**Figure 4:**
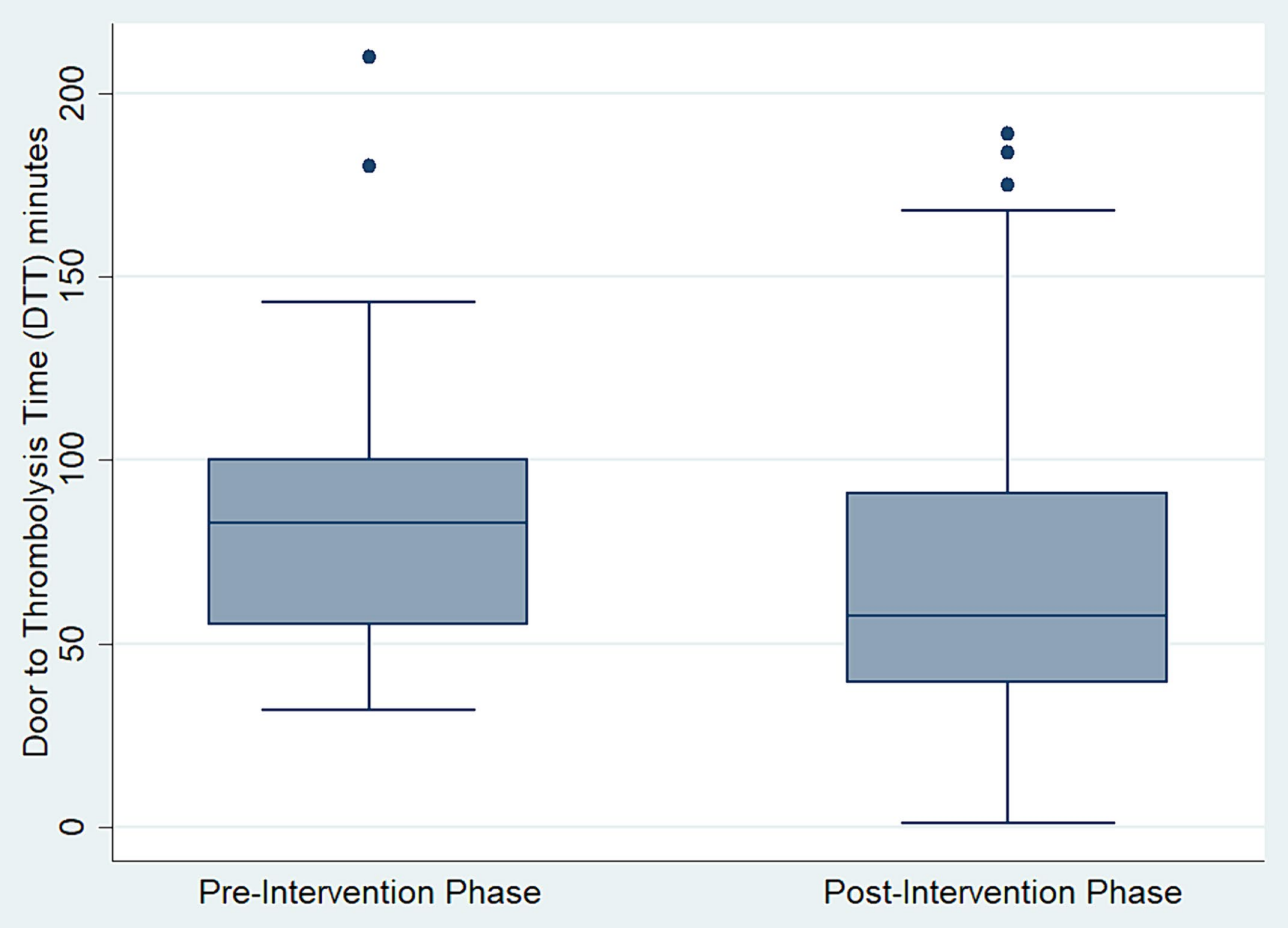
Time from door to thrombolysis comparison between pre-and post-Intervention phase.

In the pre-and post-intervention stage, 206 (45.90%) vs. 261 (55.5%) (*p* = 0.003) patients were diagnosed with a probable or confirmed ischaemic stroke. Following the study intervention, significantly more patients were treated with thrombectomy in the post-intervention phase compared to the pre-intervention phase (26/206, 12.6% vs. 54/261 20.7%, *p* = 0.022), but not thrombolysis (pre-intervention 49/206 23.8% vs. post-intervention 55/261, 21.1%, *p* = 0.484). Of the patients who received thrombolysis during the study period, those seen in the post-intervention phase were treated significantly faster with a median DTT reduction of 25 min (median DTT was 58 min in the post-intervention phase, IQR: 39, 91 vs. the pre-phase median DTT of 83 min, IQR: 55–100, *p* = 0.0324, Figure 4). The main benefit was seen in the patients’ reduction of the door to treatment, where 4 patients were thrombolysed within 30 min of regional hospital arrival in the pre-intervention phase, and no patients were thrombolysis within 30 min after arrival to the regional hospital in the pre-phase. Of the patients with a DTT over 90 min, there was no considerable difference between the phases (pre-17, post-14).

More patients underwent brain imaging during the post-intervention phase compared to the pre-intervention phase (337,71.7%, vs. 295,65.7%, *p* = 0.021), reflecting protocol awareness pre-intervention telehealth education and increased access to timely CT scanning. Furthermore, patients seen during the post-intervention phase had significantly shorter door-imaging times [pre-intervention median 35 min (IQR 18–70) vs. 49 min (IQR 25–102; *p* < 0.0001)]; 72 patients [16.4%_ were scanned within 15 min, and 138 patients (29.4%) were scanned within 30 min post-intervention, compared with 42 (9.4%) and 89 (19.8%) respectively]. Further, a reduction was seen in very long arrival to scan times, 58 (12.3%) patients with a door-scan time > 90 min post-intervention vs. 86 (19.2%) pre-intervention (*p* = 0.005). Time from regional hospital presentation to thrombectomy is quite long in South Australia due to the distances between towns and hospitals, as well as the limited infrastructure for patient transfers. During the study period, there was a trend to shorter time to thrombectomy in the post- intervention (median time to thrombectomy was 450 min, IQR: 353, 753 min, mean 706 min, and the range between 265 to 1,360 min) compared to the pre-intervention phase (median time to thrombectomy was 595 min, IQR: 532, 815 min, mean 717 min, and the range between 403 to 1725 min), however, the difference did not reach statistical significance (*p* = 0.9896).

During the study period, there were no changes in the time taken for the remote clinician assessment (the median was 30 min in the pre-intervention phase vs. 30 min in the post-intervention phase, *p* = 0.906). However, there was a trend towards a higher number of transfers being avoided during the post-intervention phase upon independent case review (206, 45.9% in the pre-intervention phase vs. 251, 53.4% in the post-intervention phase *p* = 0.271); however, did not reach significant difference. The total number of transfers between the study phases was similar (135, 30.6% in the pre-intervention phase vs. 135, 28.7% in the post-intervention phase, *p* = 0. 707), reflecting an increased stroke call volume and in-hospital recognition of stroke. Additionally, the mechanism of transfer changed from the road (44 /135, 32.6%, patients in the pre-intervention phase, 37/135, 27.4% patients in the post-intervention phase, *p* = 0.353) to air (55/135, 40.7%, patients in the pre-intervention phase, and 80/135, 59.3%, patients in the post-intervention phase, *p* = 0.002).

## Discussion

In this study, we compared the acute telestroke workflow metrics before and after a comprehensive intervention package consisting of improved telehealth technology, education and ongoing quality improvement feedback to sites. Our study found that a higher portion of patients who received thrombectomy in the post-intervention phase had shorter door-to-scan time and significantly faster door-to-thrombolysis time compared to the pre-intervention phase. The support of a seamless tele-stroke platform supported patient care, enhanced quality service improvement activities, and improved access to facilities for regional hospitals, such as imaging. Taken together, the use of education, implementation support and optimised technology led to an overall improved quality of care for patients presenting to regional hospitals in South Australia and the Northern Territory.

Previous studies have shown that telehealth programs improve stroke care in regional areas (7, 13, 14). However, significant challenges still exist in providing regional, low-volume stroke centres with adequate specialist cover with telehealth programs. In the post-intervention phase, the South Australian Tele-Stroke Service (SATS) had more participating regional hospitals using telehealth programs to access acute suspect stroke patients with neurologist consultation. Our study showed that delivering ongoing education and enabling clinicians access to vital acute stroke patient data improved the acute stroke workflows and led to improved time to reperfusion for regional patients. Importantly, during the study, there were changes to the patient care pathway, seen with the increased use of air retrievals. While this change to air-based retrieval did not show a decrease in the time to thrombectomy, it likely contributed to the increased access to thrombectomy for the regional centres. Lastly, the shift to an air-based retrieval model highlights the significant distances that patients have to travel in order to access thrombectomy services in regional South Australia and the Northern Territory.

A limitation of this study was the retrospective data Research Topic for the control period; Information on patients’ outcomes was limited due to the limited record of patients’ follow-up information and challenges in contacting patients after repatriation. Additionally, part of the study was conducted during the COVID-19 pandemic and our study result and the ability to follow-up patients was impacted. Lastly, during the study period, there may have been other quality improvement processes taking place, which may have contributed to the study results.

## Conclusion

The intervention of an optimised and customised workflow for telehealth in stroke, with ongoing quality improvement feedback, led to significant improvements in health service delivery from reperfusion treatments for patients presenting to hospitals in regional South Australia and the Northern Territory which have traditionally had limited access to acute stroke care.

## Data availability statement

The datasets presented in this article are not readily available because anonymized data not published within this article will be made available by request from qualified investigators whose proposal of data use has been approved by an independent review committee. Requests to access the datasets should be directed to AB email: abivard@unimelb.edu.au.

## Ethics statement

The studies involving humans were approved by the Central Adelaide Local Health Network Human Research Ethics Committee (CALHN HREC; CALHN Reference Number: 19347). The studies were conducted in accordance with the local legislation and institutional requirements. Written informed consent from the patients/participants or patients/participants' legal guardian/next of kin was not required to participate in this study in accordance with the national legislation and the institutional requirements.

## Author contributions

CK: Data curation, Writing – original draft, Writing – review & editing, Conceptualization, Methodology, Project administration. CC: Data curation, Formal analysis, Writing – original draft, Writing – review & editing. MaW: Conceptualization, Data curation, Validation, Visualization, Writing – review & editing. AT: Data curation, Investigation, Methodology, Writing – review & editing. JM: Data curation, Investigation, Methodology, Writing – review & editing. MiW: Conceptualization, Data curation, Investigation, Methodology, Writing – review & editing. JH: Data curation, Formal analysis, Methodology, Visualization, Writing – review & editing. JE: Data curation, Project administration, Writing – review & editing. KD: Data curation, Project administration, Writing – review & editing. BP: Data curation, Project administration, Writing – review & editing. AB: Conceptualization, Data curation, Investigation, Methodology, Project administration, Resources, Supervision, Visualization, Writing – review & editing. MP: Resources, Visualization, Writing – review & editing. SD: Resources, Supervision, Writing – review & editing. GD: Supervision, Visualization, Writing – review & editing. JJ: Data curation, Investigation, Methodology, Writing – review & editing. TK: Conceptualization, Data curation, Investigation, Project administration, Resources, Supervision, Visualization, Writing – review & editing.
